# Effect of ambient conditions in friction surfacing

**DOI:** 10.1007/s40194-024-01865-8

**Published:** 2024-11-15

**Authors:** M. Hoffmann, E. A. Duda, P. Aspes, B. Klusemann

**Affiliations:** 1 Solid State Materials Processing, Institute of Material and Process Design, Helmholtz-Zentrum Hereon, Geesthacht, Germany; 2 Laboratório de Metalurgia Física, LAMEF, Universidade Federal Do Rio Grande Do Sul, UFRGS, Porto Alegre, RS Brazil; 3 Institute for Production Technology and Systems, Leuphana University Lüneburg, Lüneburg, Germany

**Keywords:** Friction surfacing, Preheating, Underwater, Microstructure, Precipitate, Hardness

## Abstract

Friction surfacing (FS) is a solid-state deposition process in which layers are deposited on a substrate surface by frictional heat and severe plastic deformation of a consumable stud material below its melting temperature. Bonding occurs due to accelerated diffusion. The deposition of several layers on top of each other is referred to as multi-layer FS (MLFS), a promising candidate for additive manufacturing (AM) as it offers advantages over fusion-based AM. In this study, the MLFS process for the precipitation-hardenable alloy AA2024 is investigated regarding the influence of environmental process conditions, i.e., preheating of the substrate like other AM processes as well as underwater and room temperature experiments. The influence of ambient conditions on the process behavior, the layer geometries, the microstructure, and the mechanical properties is shown. Preheating the substrate leads to an overall higher process temperature (424.1 °C), resulting in thinner and wider layers, larger grains, an overaged microstructure, and a smooth hardness transition in the MLFS stacks from top (140 HV0.1) to bottom (95 HV0.1). The lower the process temperatures, e.g., for underwater FS (326.5 °C), the thicker and less wide the layers and the smaller the grains. The hardness shows a periodic pattern at the layer interface, which is more pronounced at lower process temperatures, i.e., the hardness values range from 100 HV0.1 to 150 HV0.1.

## Introduction

The solid-state deposition process friction surfacing (FS) enables the formation of layers by means of frictional heat and severe plastic deformation of a consumable stud while experiencing an axial force, a rotational speed, and a translational movement. Bonding of the layer is achieved due to accelerated diffusion processes, which allow the combination of a wide range of metallic materials, such as steel [1], aluminum [2], titanium [3], or Inconel [4] as well as dissimilar materials, e.g., aluminum and steel [5] or titanium and steel [6]. The applications of FS range from repairing tasks, such as crack filling [7] and improving wear properties [8], to additive manufacturing (AM) [9]. The FS variant in which several layers are deposited on top of each other is referred to as multi-layer FS (MLFS). Due to the solid-state nature of the MLFS process, it can overcome some of the major disadvantages of fusion-based AM processes like porosity, cracking, high residual stresses, or inhomogeneous microstructures [10, 11]. To avoid some of these defects also in fusion-based AM processes, preheating the substrate plate to decrease the temperature gradient and cooling rates between the layers generated and the substrate is a commonly used approach [12, 13]. However, with some materials, such as aluminum alloys, preheating the substrate plate can lead to an in situ overaging of the deposited layer materials that can result in lower strength and larger grain sizes compared to colder substrate plates [14]. On the other hand, cooling the stud material during the FS process enhances the deposition efficiency, i.e., the ratio between deposited material and total consumed stud material [15]. For friction stir welding (FSW), joining two aluminum alloys underwater leads to several differences compared to conventional FSW, like a different thermal cycle with a lower maximum temperature and higher heating and cooling rates [16]. This caused lower precipitate coarsening [16], which eventually results in better mechanical properties, like higher tensile strength and elongation [17] as well as a narrower softening zone [18]. Furthermore, underwater FSW can lead to a reduction in residual stresses [19, 20].

In general, the thermal history in precipitation hardenable alloys such as AA2024 plays an important role in the development of mechanical properties. For AA2024, the ratio of Cu to Mg leads to different precipitation sequences. A low Cu:Mg ratio, i.e., ~ 1:1 [21], leads to the sequence of solid solution $\left(SS\right)\to$ Guinier–Preston-Bagaryatsky zones $\left(GPB\right)\to {S}^{{\prime}{\prime}}\to {S}^{\prime}\to S$ (Al_2_CuMg) [22, 23], whereas higher Cu:Mg ratios result in $SS\to$ Guinier–Preston zones $\left(GP\right)\to {\theta }^{{\prime}{\prime}}\to {\theta }^{\prime}\to \theta$ (Al_2_Cu) [24]. For intermediate ratios of Cu to Mg, both sequences are present simultaneously [25]. Ehrich et al. [26] investigated the precipitation in FS for AA2024 both in the layer and the heat-affected zone (HAZ) of the substrate. The results showed that the HAZ can be divided into three different subregions, depending on the process temperature experienced. At high process temperatures close to the substrate surface, the volume fraction of GP/GPB and ${S}^{\prime}/{\theta }^{\prime}$ as well as the hardness decreases (HAZ1). The highest hardness is observed in HAZ2, which is located below HAZ1. This is due to medium temperatures, increasing the volume fraction and size of ${S}^{\prime}/{\theta }^{\prime}$ as well as the volume fraction of GP/GPB. In comparison, HAZ3, which is furthest away from the substrate surface, exhibits the lowest temperatures, resulting in a decrease in the volume fractions of GP/GPB and ${S}^{\prime}/{\theta }^{\prime}$ as well as the hardness. Coarse, overaged $S/\theta$ are reported in the deposited layer.

The aim of this study is to investigate three cases, i.e., preheating of the substrate, performing the FS process at room temperature (RT), and enhancing the cooling by performing the FS process underwater. The influence of the change of the ambient conditions on the MLFS process in terms of layer geometry, process temperature, and deposition behavior, i.e., process torque and stud consumption rates, microstructure, and hardness is studied using the precipitation hardenable alloys AA2024.

## Materials and methods

Each experiment was performed using a machine designed especially for FS experiments (RAS, Henry Loitz Robotik, Germany), which provides a working area of 1.5 × 0.5 m^2^. It allows maximum axial forces, rotational speeds, and spindle torques of 60 kN, 6000 rpm, and 200 Nm, respectively. Process data, such as forces and displacements in *x*-, *y*-, and *z*-direction as well as rotational speed and spindle torque are recorded during the process. The selected materials and process parameters are summarized in Table 1. The parameters were selected based on a previous study [27], to deposit 200-mm-long MLFS stacks in force-control mode.

**Table 1:** Materials and process parameters used in this study

Parameter	Description/value
Stud material	AA2024-T351
Stud geometry	20 mm (diameter) × 100 mm (length)
Substrate material	AA2024-T3
Substrate thickness	7.5 mm
Axial force	8 kN
Rotational speed	800 rpm
Travel speed	6 mm/s

**Fig. 1:**
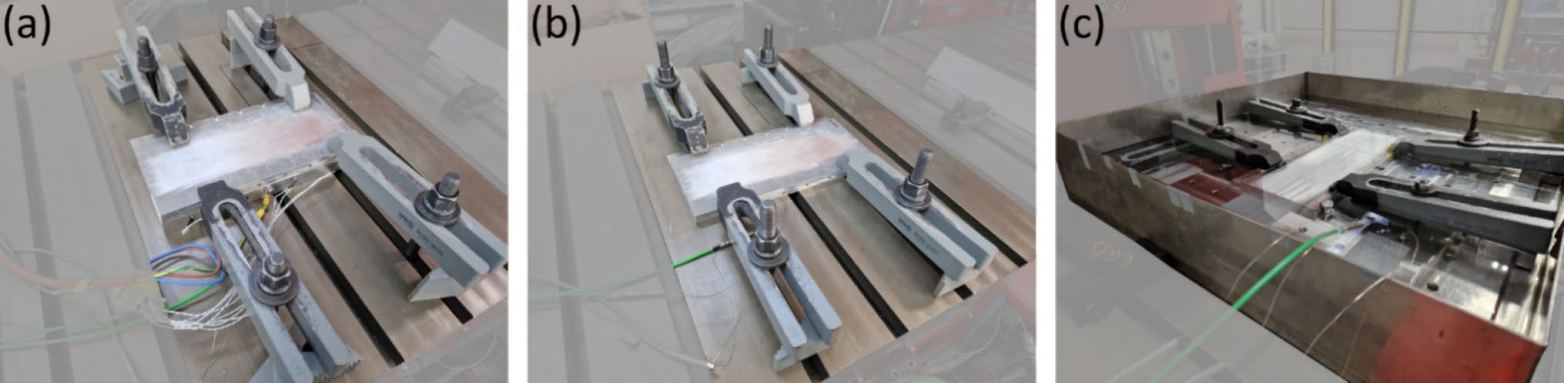
Experimental setup of preheating (**a**), RT (**b**), and underwater (**c**) FS process

The ambient conditions during the process have been altered by changing the experimental setup as shown in Fig. 1. These conditions are investigated: the substrate was preheated to 200 °C, the initial temperature of the substrate was RT, i.e., approximately 20 °C, and the whole process took place underwater in a basin with a water temperature of approximately 20 °C. The water level was selected to be 2 mm above the substrate and layer surface, respectively. In each condition, a five-layer MLFS stack has been built. As a backing between the substrate and the machine table, an 8-mm AA2024 plate was used for the RT and underwater experiments. For the preheating deposits, a 25-mm-thick plate was selected, in which four heating cartridges with a heating power of 400 W each were inserted. To determine the temperature near the interface between layer and substrate, five type K thermocouples were inserted into holes in the substrate from the bottom side to a distance of 0.5 mm from its top surface. Thermal paste was used to avoid air gaps between the thermocouple tips and the substrate. The distance between the thermocouples orthogonal to the travel direction of the stud was 5 mm, with the thermocouple in the center being aligned with the deposition path. The data acquisition frequency was set to 50 Hz. From the temperatures, heating and cooling rates were determined.

From each MLFS stack, three cross-sections were cut. Two of them were embedded and prepared by grinding and polishing as well as macroscopically captured and geometrically analyzed by VHX-6000 digital microscope (Keyence, Germany). For the geometrical analysis, the thickness and the width of the layers were determined. These measures together with the stud consumption rate allow to do the analyses as proposed by Fukakusa [28] for the assumed contact radius of the stud tip during the process. According to Fukakusa, the assumed contact radius ${{\varvec{r}}}_{{\varvec{c}}}$ is calculated using:

1 $${r}_{c}=\sqrt{\frac{wtv}{\pi {v}_{z}}},$$

with layer width $w$, layer thickness $t$, travel speed $v$**,** and stud consumption rate ${v}_{z}$. The volumetric deposition rate $D{R}_{\text{vol}}$ is calculated via:

2 $$D{R}_{vol}=wtv,$$

the volumetric stud consumption rate $C{R}_{\text{vol}}$ is calculated via:

3 $$C{R}_{vol}=\pi {r}^{2}{v}_{z},$$

with stud radius $r$. Dividing $D{R}_{\text{vol}}$ by ${CR}_{\text{vo}l}$ results in the deposition efficiency ${\eta }_{\text{deposition}}$ defined by Gandra et al. [29] as follows:

4 $${\eta }_{deposition}=\frac{D{R}_{vol}}{C{R}_{vol}}=\frac{wtv}{\pi {r}^{2}{v}_{z}}$$

Inserting the square of Eq. (1) into Eq. (4) shows that the deposition efficiency can also be calculated by the contact radius:

5 $${\eta }_{deposition}={\left(\frac{{r}_{c}}{r}\right)}^{2}$$

The deposition efficiency describes the percentage of consumed stud material that is deposited into the layer. The rest of the stud material forms the FS typical flash.

The two samples prepared were then hardness tested using a DuraScan 70 G5 (EMCO-Test Prüfmaschinen GmbH, Germany) and investigated by electron backscatter diffraction (EBSD) analysis, respectively. For the hardness mappings, indentations were performed in accordance with DIN EN ISO 6507 using a 136° Vickers indenter at a load of 0.1 kg in a pattern with a 0.2-mm distance. The EBSD scans were conducted at an FEI Quanta FEG 650 scanning electron microscope (Thermo Fisher Scientific Inc., USA) with an EDAX Velocity Series EBSD detector (AMETEK Inc., USA). The scans were performed in the third layer of each MLFS stack, using a beam voltage of 15 kV, a step size of 0.2 µm, and a working distance of 17 mm as scan parameters. The MATLAB (The Mathworks Inc., USA) toolbox MTEX 5.8.1 was used for the EBSD data analysis. The minimum grain size was set to 3 pixels and the grain boundary misorientation threshold to 2°.

The third cross-section was used to investigate the formation and dissolution behavior of precipitates in the MLFS stacks using differential scanning calorimetry (DSC). Samples from the first and fifth layers of each MLFS stack were cut from this cross-section. For the DSC analysis, the DSC 200 F3 Maia differential scanning calorimeter (Netzsch, Germany) was used and operated at a heating rate of 20 K/s. All samples were placed in Al_2_O_3_ crucibles. A pure Al sample was measured to determine the baseline correction. The post-processing of the data was based on the method described by Osten et al. [30].

## Results and discussion

### Layer geometry and temperatures

**Fig. 2:**
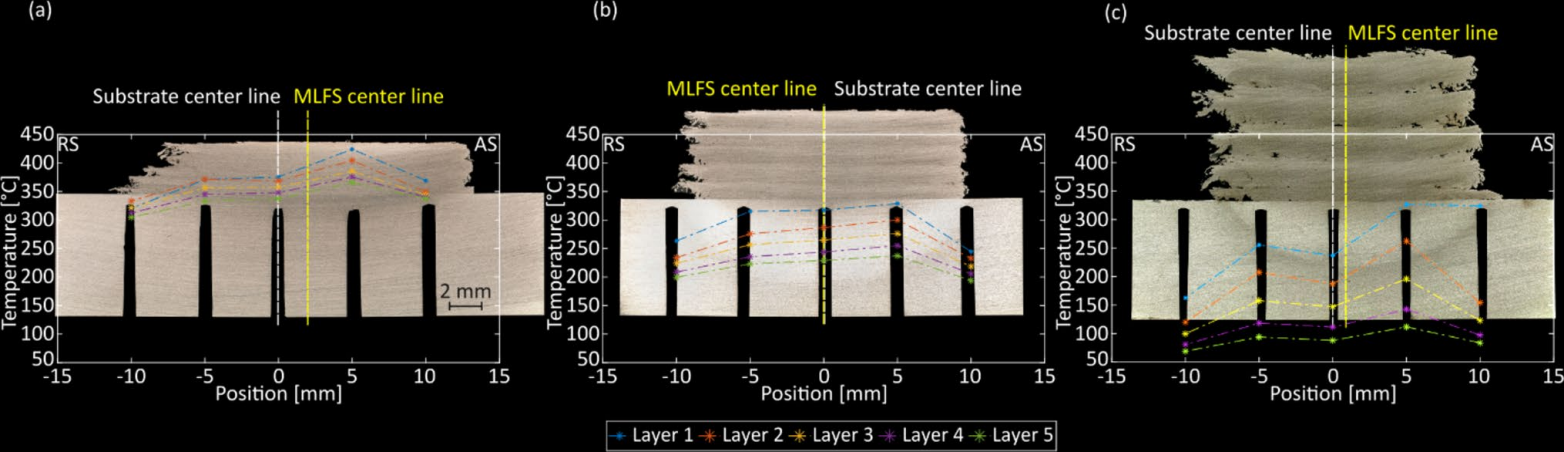
Cross sections and interface temperatures of preheating (**a**), RT (**b**), and underwater (**c**) MLFS stacks

The layer geometries in the three MLFS stacks are influenced by the ambient process conditions as shown in Fig. 2. The average layer thicknesses increase from 0.67 mm for preheating to 1.21 mm for RT to 1.93 mm for the underwater experiments. At the same time, the average layer width decreases from 23.68 mm for preheating to 20.38 mm for RT and to 19.76 mm for underwater FS. Besides that, the temperature measurements show that the ambient process conditions play a significant role in the maximum process temperature. The highest temperature prevails in the preheating condition, i.e., 424.1 °C, whereas the coldest temperature was measured for the underwater experiment, i.e., 326.5 °C. For all three cases, the highest temperature was measured on the advancing side (AS), which indicates a shift of the stud and therefore the whole layer towards this side as shown in Fig. 2b and c. This phenomenon is often reported in the literature for FS [31]. In the preheating case, the difference between the thermocouple on the AS and the center is the highest, which is caused by the softening of the substrate due to the preheating. The softer substrate therefore is deformed during the process above the tip of the thermocouple (Fig. 2a), leading to a better measurement contact.

**Fig. 3:**
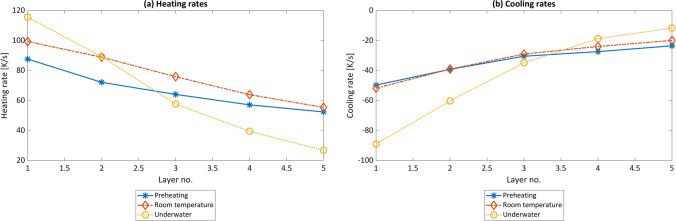
Heating (**a**) and cooling rates (**b**) of all MLFS stack layers calculated from the temperature data measured during the FS process

From the temperature measurements, besides the maximum temperature, heating and cooling rates were calculated and are displayed in Fig. 3. In the case of underwater FS, both the heating and the cooling rates are highest in the first layers, which is due to the larger cooling of the surrounding water. In the higher layers, the maximum temperature is significantly lower with increasing distance between heat source and measurement position, so that the heating and cooling rates decrease as well. In the experiments with preheating and at RT, the heating and cooling rates were lower. This could be related to the fact that the temperature difference between the maximum process temperature and the initial and final substrate temperature is lower in these two process conditions. If only the first layer is considered, the highest heating and cooling rates are measured in the underwater FS, while the lowest rates are present in the preheating experiment.

The change of layer geometry with temperature is in accordance with the findings of Kallien et al. [32], where a higher temperature results in a wider and thinner FS layer. Based on Eq. (1), the contact radius of the stud tip during the FS process can be calculated with the layer geometry information. Since the percentage change in layer thickness caused by changes in the ambient conditions of the process is the greatest, this also has the greatest influence on the calculation of the assumed contact radius. Therefore, due to the lowest layer thickness of the preheating FS, the assumed contact radius is the lowest, i.e., 5.21 mm. It increases for RT FS to 5.87 mm and for underwater FS to 6.89 mm. Furthermore, the increase in layer thickness results in a higher deposition efficiency, i.e., the ratio of deposited material to consumed stud material. The deposition efficiency is at 27.15% (preheating) and increases up to 34.43% (RT) and 47.42% (underwater). These results agree with the one reported by Krohn et al. [15], where an increase in the deposition efficiency using water-spray cooling compared to RT experiments for AA6082 stud material was observed. In the underwater case, however, the bonding width decreases, which is evident from the larger number of defects at the bottom of the layers (Fig. 2c). The lower process temperature and the associated higher cooling rate could lead to a poorer consolidation of the layer-forming material on the substrate or the underlying layers. The underwater FS process results in a sufficiently high cooling efficiency, which cools the layer material to be deposited to such an extent that proper deformation of the material and consolidation towards the substrate under the axial pressure of the stud material is hindered. As a result, the material is poorly bonded to the substrate. Therefore, better bonding might be achieved by reducing the cooling rate in underwater FS or increasing the process temperature. A lower cooling rate could be achieved by increasing the water temperature, and a higher process temperature by changing the process parameters, e.g., increasing rotational speed or axial force. However, the deposition efficiency is expected to be reduced at the expense of better layer consolidation.

### Process behavior

**Fig. 4:**
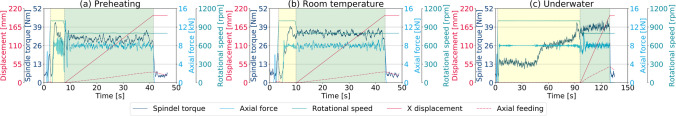
Process data recorded during the FS process of the third layer of preheating (**a**), RT (**b**), and underwater (**c**) FS. The highlighted areas are the plasticizing (yellow) and the deposition phases (green)

Besides the layer geometry, differences are discernible for the process behavior, i.e., mainly the duration of the plasticizing phase^1^ and the measured process torque at the spindle of the FS machine. Figure 4 shows the measured process data during the FS process, i.e., axial force, spindle torque, rotational speed, and displacements in *x*- and *z*-direction. As the deposition phase for all the processes has the same duration, the significantly longer process duration of the underwater FS results from the longer plasticizing phase. During this phase, the stud material is heated up by the friction between the stud tip and the substrate surface. As more heat is dissipated in the part of the stud that is underwater, the stud takes longer to heat up sufficiently.

**Fig. 5:**
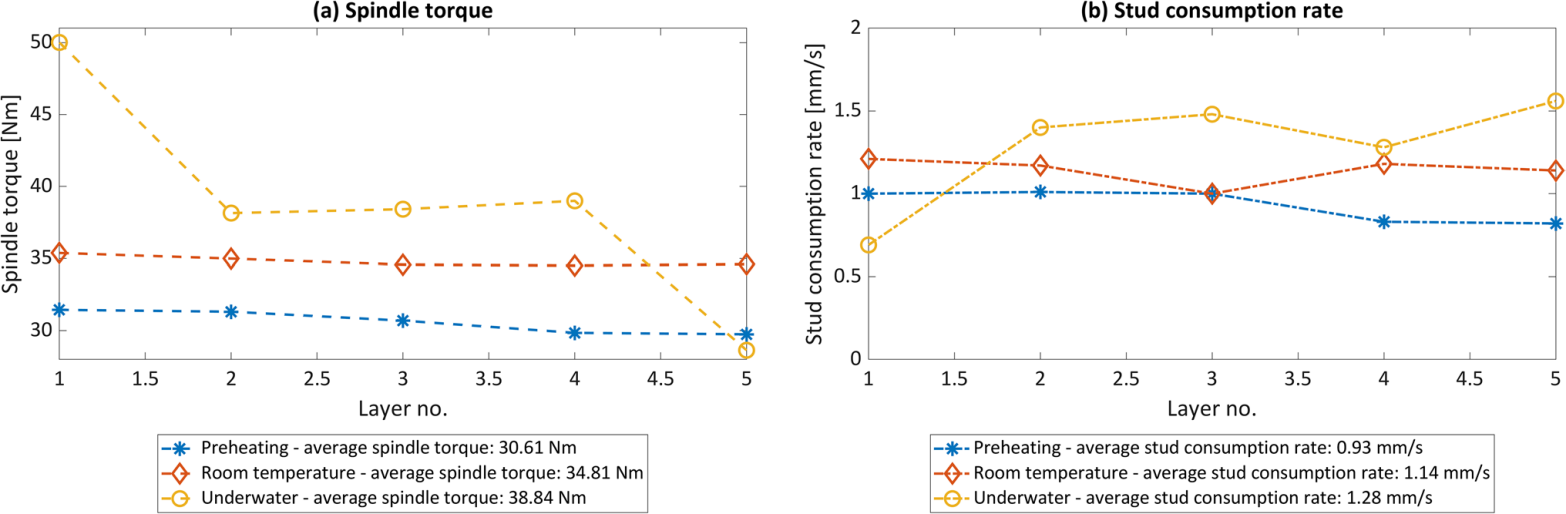
Spindle torque (**a**) and stud consumption rate (**b**) of all MLFS stack layers

As shown in Fig. 5a, higher spindle torque was reached for lower process ambient temperatures. As the colder ambient process conditions underwater represent a greater heat sink during the process, the material at the stud tip remains at a lower temperature and therefore obtains a higher resistance against plastic deformation, leading to a higher spindle torque. This indicates that higher energy input is required to deposit material underwater, as the process energy input is directly related to the torque. In addition, the spindle torque decreases with increasing number of layers, which has already been described in the literature [33]. On the other hand, the five-layer average stud consumption increases from preheating to RT to underwater FS (Fig. 5b). The stud consumption rate is calculated during the deposition phase, i.e., between the start and the end of the translational movement, and the time of this phase. As the average initial stud plasticizing phase in the underwater condition lasts longer compared to RT and preheating FS, i.e., 38.75 s (underwater), 8.14 s (RT), 5.99 s (preheating), a larger area of the stud close to its tip heats up and thus the material becomes softer, resulting in a higher stud consumption.

### Microstructure

**Fig. 6:**
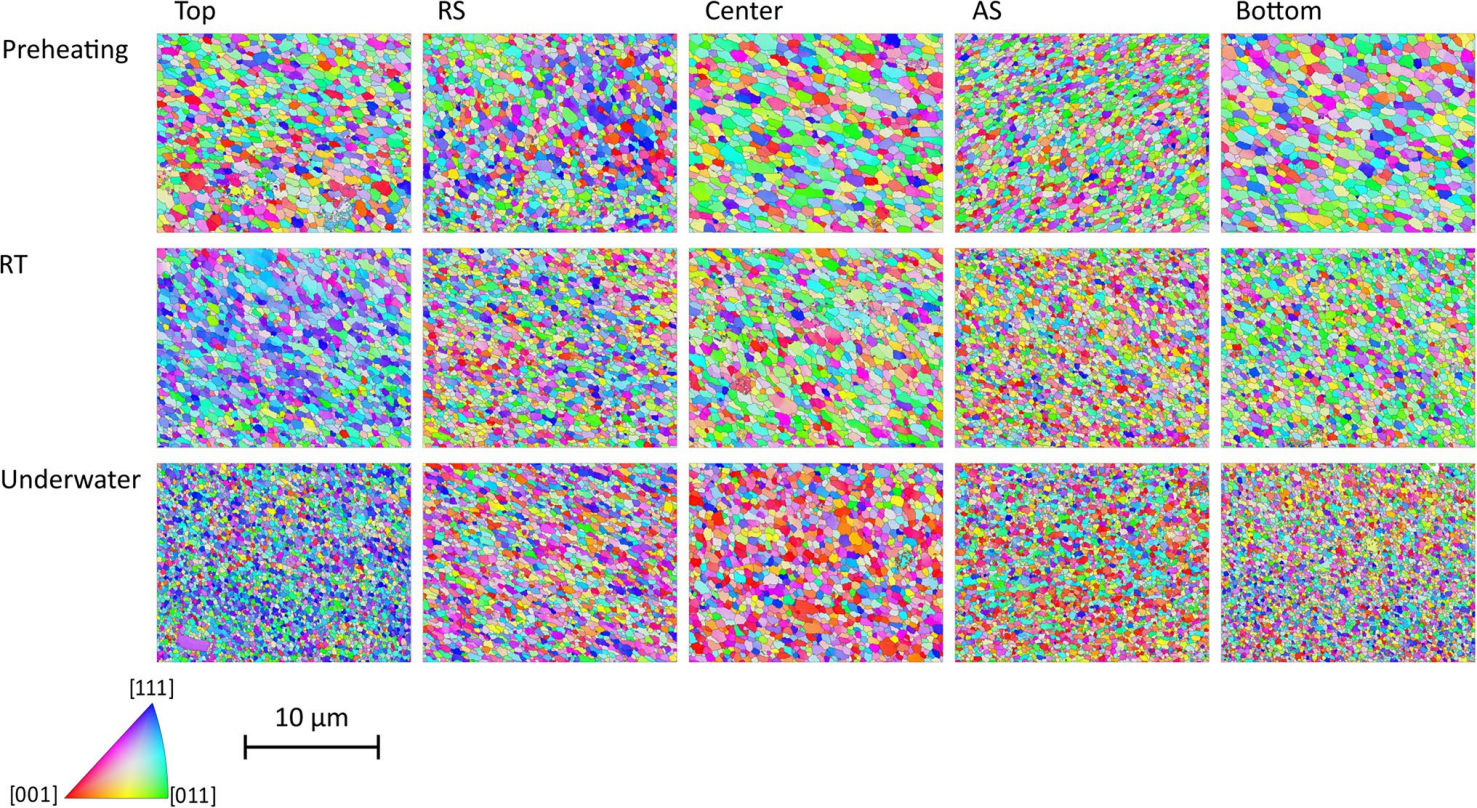
IPF-Maps of the EBSD scans in five different scan areas of the three different ambient process conditions; enlarged by the factor 9. Low-angle grain boundaries are displayed in gray and high-angle grain boundaries in black

In terms of microstructure, five different areas of the third layer were investigated, i.e., AS, retreating side (RS), center, bottom, and top. The corresponding IPF-Maps are displayed in Fig. 6.

**Fig. 7:**
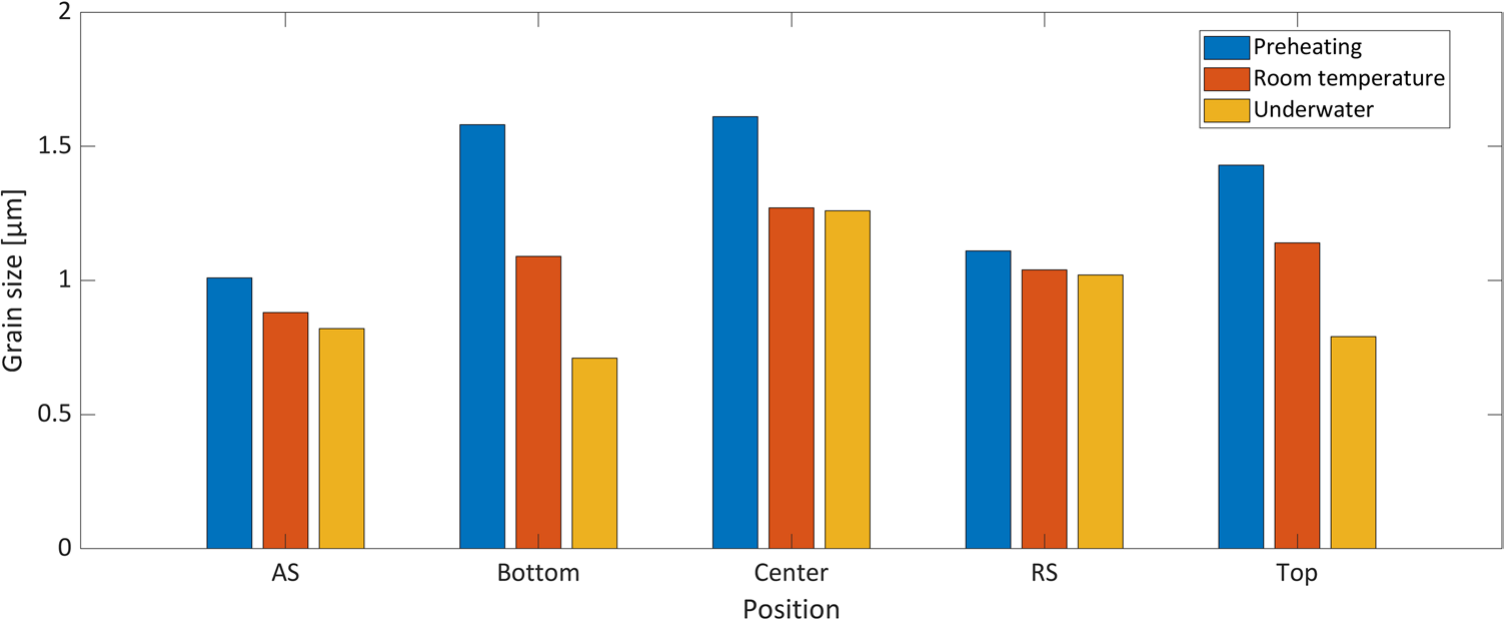
Grain size histogram for all areas, i.e., AS, RS, center, bottom, and top, of preheating (**a**), RT (**b**), and underwater (**c**) MLFS stacks

From all areas scanned, the grain size is the largest for substrate preheating and the smallest for underwater FS (Fig. 7) and therefore correlates with the achieved maximum temperature during the deposition process. Often a decrease of grain size from the top to the bottom is reported in literature like for the AA2024 deposits of Rahmati et al. [34] due to an enhanced heat flow to the substrate. However, Kallien et al. [27] showed for AA2024 as well as AA5083 stud material that the grain size distribution follows a periodic pattern with smaller grains in the bottom and top part of the layer due to higher cooling rates in the bottom part and higher strain rates in the top. In the present study, the same behavior is observed, with the largest grains in the center of the layer. For preheating and RT FS, the AS shows the smallest grains, whereas in underwater FS, the smallest grains are observed at the bottom of the layer. As often mentioned in the literature, the material flow during the FS process is complex [35], influencing local strain rates [36]. Due to the different process temperatures between the cases presented here, the local strain rate can also change. This leads to slight changes in the grain sizes in the cross-section of the layer; therefore, the smallest grain sizes are not always present on the AS. Furthermore, for the underwater FS, both substrate and water act as a heat sink and increase the cooling rates, which results in the finest grains overall. Both the process temperature and the local cooling rates appear to influence the final grain size when the same process parameters are applied.

**Fig. 8:**
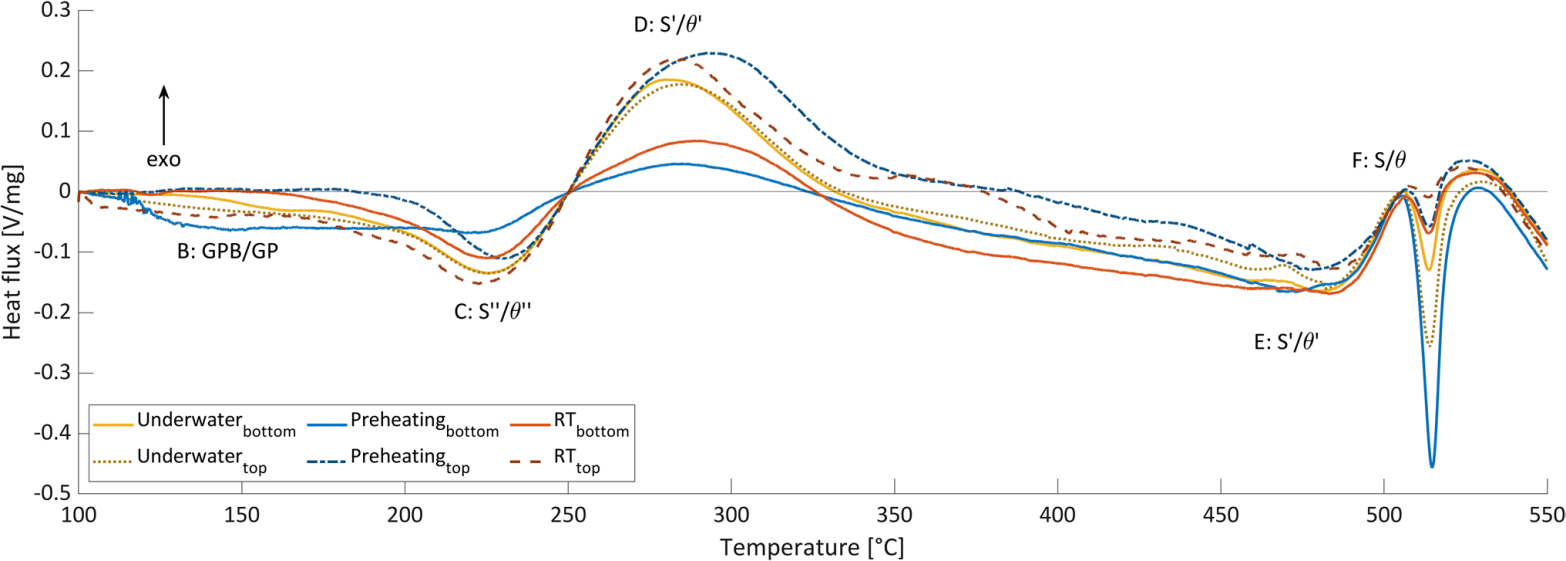
DSC curves for bottom and top region of preheating, RT, and underwater MLFS stacks

To compare the influence of the ambient conditions as well as the position, i.e., bottom and top layer of the MLFS stacks, on the precipitation formation during the MLFS process, DSC curves were recorded (see Fig. 8). For the underwater FS, no significant differences between the DSC curves in the top and bottom parts of the MLFS can be seen. At RT, the bottom part shows lower peaks B (GPB/GP dissolution), C (${S}^{{\prime}{\prime}}/{\theta }^{{\prime}{\prime}}$ dissolution), and D (${S}^{\prime}/{\theta }^{\prime}$ formation), which means that less GPB/GP and ${S}^{{\prime}{\prime}}/{\theta }^{{\prime}{\prime}}$ as well as more ${S}^{\prime}/{\theta }^{\prime}$ already existed in the deposited layer after the process. Furthermore, the higher peak E indicates that during the DSC measurement, more ${S}^{\prime}/{\theta}^{\prime}$ is dissolved. A reason why ${S}^{\prime}/{\theta }^{\prime}$ has not been dissolved or transformed to $S/\theta$ during the process is the maximum FS process temperature of 328.9 °C, which is not sufficiently high enough and more likely causes a ${S}^{\prime}/{\theta }^{\prime}$ coarsening, as also reported for ${\theta }^{\prime}$ coarsening in the HAZ during refill friction stir spot welding of AA2219 [37]. Also, the higher peak F indicates that $S/\theta$ is formed during the DSC and not during the FS process. A preheating of the substrate results in similar differences between the bottom and the top as for the RT curves. The only difference comparing RT and preheating FS is the behavior between 100 and 250 °C. In the bottom part, peak B exists, which means that GPB/GP dissolved during or after the process due to the constant substrate temperature of 200 °C and re-precipitated during the post-process natural aging. In the top part, however, the peak C is more pronounced, i.e., in this layer, more of the strengthening precipitates ${S}^{{\prime}{\prime}}/{\theta }^{{\prime}{\prime}}$ exists.

Furthermore, the two different regions have been compared for the three processing conditions. In the bottom part, for the preheating FS, lower peaks C and D were detected, i.e., the least ${S}^{{\prime}{\prime}}/{\theta }^{{\prime}{\prime}}$ and the most ${S}^{\prime}/\theta ^{\prime}$ were present. In contrast, the underwater FS shows the highest peaks C and D for all samples from the bottom, and thus the opposite behavior as for the preheating case is observed. In the top part, the ambient condition seems to have less influence on the precipitation formation, which is due to the different thermal histories of the layer at the top. The layer in the bottom undergoes additional thermal cycles during the deposition process of the overlying layers, which has a significant influence on the precipitate formation. Overall, the underwater FS seems to lead to a more homogeneous microstructure in terms of precipitation formation comparing bottom and top layers of the MLFS stacks. This is due to the lower process temperatures and the higher heating and cooling rates, which result in a shorter temperature exposure compared to the other two ambient conditions. In preheating FS, the lower heating and cooling rates mean that the material is exposed to higher temperatures for longer. This results in overaging of ${S}^{\prime}/{\theta }^{\prime}$ in the bottom part of the MLFS stack due to coarsening.

### Hardness

**Fig. 9:**
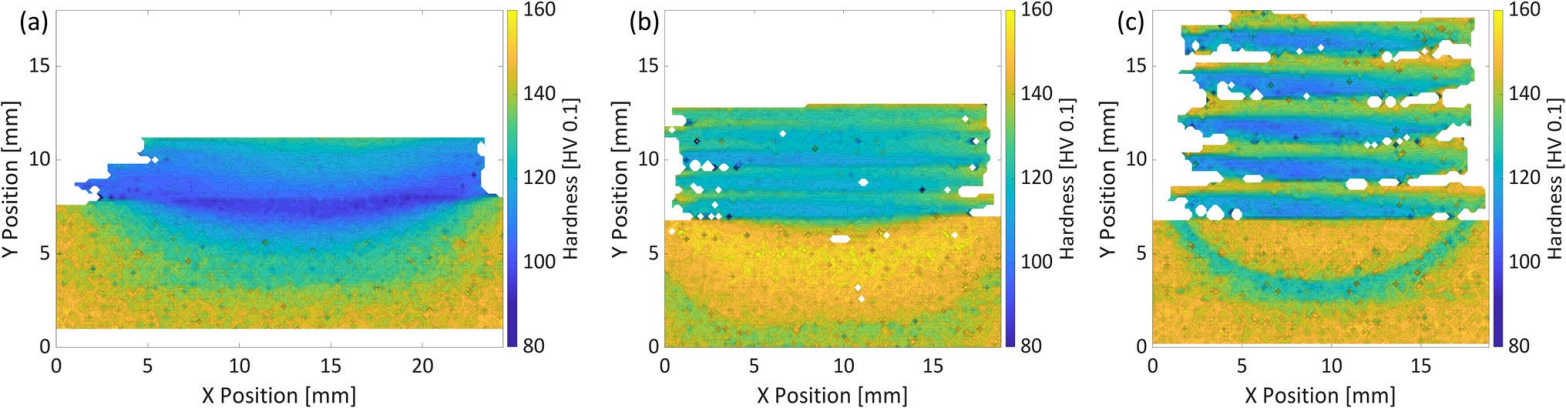
Hardness mapping of preheating (**a**), RT (**b**), and underwater (**c**) MLFS stacks

The hardness measurements reveal significant differences between the preheating case MLFS, and the ones built at RT and underwater (Fig. 9), which is related to the precipitation formation as discussed in the section before. While both RT and underwater MLFS show an almost periodic hardness pattern from the top of the stack towards the layer-substrate-interface with higher hardness values in the top of the layers, i.e., 150 HV0.1, and the lower hardness in the center of the layer, i.e., 100 HV0.1, the MLFS stack for the preheating case shows a smooth hardness transition from the top of the stack, i.e., 140 HV0.1, towards the bottom. The lower maximum hardness for the preheating MLFS stack might be due to the lower content of GPB/GP compared to the RT and underwater MLFS stack. The decrease of the hardness in the center of the layer is related to the slightly larger grains, as also reported for the non-precipitation-hardenable alloy AA5083 in an MLFS investigation by Kallien et al. [27].

The lowest hardness of 95 HV0.1 for the preheated MLFS is measured in the interface of the layer to the heat-affected zone (HAZ). The decrease of hardness in the MLFS stack is related to the overaging by coarsening of the ${S}^{\prime}/{\theta }^{\prime}$ precipitates as shown above. The hardness values of the RT and underwater MLFS stacks are in the same range, whereas the differences in hardness between the different layer areas are more pronounced for the underwater FS due to the thicker layers.

Also, the HAZs show the differences in hardness, comparing the preheating to the two other cases. The former obtains a HAZ in which, like the MLFS stack, the hardness transits smoothly from the lowest hardness below the interface towards substrate base material hardness of around 150 HV0.1 in the bottom part of the substrate. As the substrate was preheated to 200 °C, GPB/GP precipitates that existed in the AA2024 T3 substrate were already dissolved before the FS process. Thus, only the temperatures above 200 °C have an influence on the precipitation, i.e., dissolution of ${S}^{{\prime}{\prime}}/{\theta }^{{\prime}{\prime}}$, as well as the formation of ${S}^{{\prime}}/{\theta }^{{\prime}}$. Since higher temperatures are present in the upper region of the substrate, ${S}^{{\prime}{\prime}}/{\theta }^{{\prime}{\prime}}$ precipitates will dissolve there and ${S}^{\prime}/{\theta }^{\prime}$ will form or coarsen. In the lower, colder region of the substrate, on the other hand, only ${S}^{{\prime}{\prime}}/{\theta }^{{\prime}{\prime}}$ precipitates dissolve and the already existing ${S}^{\prime}/{\theta }^{\prime}$ remains.

In the RT and underwater FS cases, the sections of the HAZs are comparable to the ones reported by Ehrich et al. [26] for AA2024 single layer deposits, i.e., HAZ1 directly below the interface with a lower hardness due to a lower volume fraction of GPB/GP and ${S}^{{\prime}{\prime}}/{\theta }^{{\prime}{\prime}}$, the surrounding material in the harder HAZ2 (increasing amount of GPB/GP as well as ${S}^{\prime}/{\theta }^{\prime}$) and the HAZ3, which again is softer and surrounds the HAZ2. For the HAZ sizes and hardnesses, RT and underwater differ. The RT-HAZs are much wider and deeper. This might be related to the fact that the substrate is acting as the main heat sink at RT. Most of the heat is conducted through the substrate, leading to a longer temperature exposure, whereas in the underwater FS, the water acts as additional cooling. However, the hardness in HAZ3 is lower in the underwater condition. One possible explanation for this is the different temperatures in these areas at RT compared to underwater. At RT, the temperature is high enough to form ${S}^{\prime}/{\theta }^{\prime}$, while at underwater FS, the temperatures are too low to form ${S}^{\prime}/{\theta }^{\prime}$. Therefore, the microstructure at underwater FS is underaged; at RT, it is between underaged and peak aged [38] and thus HAZ3 shows a higher hardness.

## Conclusions

The present study investigated the influence of ambient processing conditions during the MLFS process of AA2024 at constant process parameters. The experiments performed underwater resulted in thicker and less wide layers that led to the highest deposition efficiency of the cases investigated. Besides that, the bonding of the layer deteriorates, which is indicated by defects at the interface between the layers for underwater FS. Furthermore, a lower process temperature was measured which led to smaller grains in all areas investigated. Due to the high cooling of the water, the process requires a higher energy input, indicated by a higher spindle torque. The precipitation investigation showed that the preheated MLFS stacks tend to be more overaged, whereas for the underwater FS, no significant differences between the top and the bottom part can be seen. The resulting hardness for the preheating case shows a transition from high hardness values in the top towards lower hardness values in the bottom part of the first layer. RT and underwater MLFS stacks show higher hardness values in the top part of the layers and lower hardness values in their centers. For the RT FS, a slight decrease of the layer-average hardness between the top and the bottom layer exists, which is not discernible for the underwater FS, where both the top and bottom layers hove similar hardness values.

## Data Availability

The obtained data from this research is online available at Zenodo (10.5281/zenodo.14033266).

## Footnotes

1 During this phase, the stud material heated up at the starting position. The translational movement was superimposed as soon as an initial stud shortening of 0.5 mm was reached. This value was selected for all three processing conditions.
